# The Immunomodulatory Potential of tolDCs Loaded with Heat Shock Proteins

**DOI:** 10.3389/fimmu.2017.01690

**Published:** 2017-11-30

**Authors:** Willem van Eden, Manon A. A. Jansen, A Charlotte MT de Wolf, Irene S. Ludwig, Paul Leufkens, Femke Broere

**Affiliations:** ^1^Department of Infectious Diseases and Immunology, Faculty of Veterinary Medicine, Utrecht University, Utrecht, Netherlands; ^2^Trajectum Pharma, Utrecht, Netherlands

**Keywords:** self-tolerance, autoimmunity, heat shock protein, regulatory T cell, peptide

## Abstract

Disease suppressive T cell regulation may depend on cognate interactions of regulatory T cells with self-antigens that are abundantly expressed in the inflamed tissues. Heat shock proteins (HSPs) are by their nature upregulated in stressed cells and therefore abundantly present as potential targets for such regulation. HSP immunizations have led to inhibition of experimentally induced inflammatory conditions in various models. However, re-establishment of tolerance in the presence of an ongoing inflammatory process has remained challenging. Since tolerogenic DCs (tolDCs) have the combined capacity of mitigating antigen-specific inflammatory responses and of endowing T cells with regulatory potential, it seems attractive to combine the anti-inflammatory qualities of tolDCs with those of HSPs.

## Introduction

Regulatory T cells (Tregs) downmodulate unwanted immune responses. The induction or expansion of Tregs with the use of antigen-loaded tolerogenic dendritic cells (tolDCs) is a novel and attractive therapeutic possibility. Tregs are predominantly immunosuppressive CD4^+^ T cells, selected in the thymus on the basis of relatively high-affinity interactions with self-antigens (1). These cells are called natural Tregs (nTregs). Alternative populations of Tregs may become induced by peripheral antigen presentation in tolerance promoting tissues leading to what is called peripheral or induced Tregs (pTreg). Comparisons between T cell receptor (TCR) repertoires of thymus-derived nTregs and gut-residing pTregs have not led to direct conclusions on the relative significance of either nTregs or pTregs. Some studies, such as those for the colon, have reported a relatively unique nature of TCRs present on gut Tregs (2), whereas others have emphasized the presence of shared TCRs between thymic and colon Tregs (3, 4). Nonetheless, in both situations, it is proposed that gut-residing Tregs expand after recognition of microbiota-associated microbial antigens. The cognate interactions with these foreign antigens would fit well with a unique nature of colon Treg TCRs, whereas a driving activity by self-cross-reactive microbial antigens would be more compatible with the shared TCR idea.

Microbial heat shock proteins (HSPs) are antigens with a well-established tolerance promoting capacity. Since the identification of mycobacterial HSP60 as the driving antigen for modulatory T cells in the adjuvant arthritis model (5), immunization with microbial HSPs, mainly HSP60 and HSP70, was shown to inhibit disease in various inflammatory models (6–8). Subsequent analysis of the specificities of the microbial antigen responding T cells led to the hypothesis that conservation of HSPs was critical in the tolerance promoting character of HSPs (9). T cells with specificity for conserved microbial sequences are cross-reactive with (mammalian) self-HSPs and therefore have potential to regulate by targeting a regulatory effect to upregulated self-HSP in inflamed tissues (6, 10). A possible sequence of mechanistic events (see Table 1) would be that T cells, or Tregs in this case, are selected in the thymus on the basis of self-HSP recognition (Table 1). For example, HSP70 is abundantly expressed in normal thymic epithelial cells (11). In addition, HSP70 epitopes were found on thymic dendritic cells (DCs) which demonstrates that these epitopes are presented in the healthy human thymus (12). For these reasons, central tolerance may be assisted by HSP-driven thymic-positive selection. In the periphery, HSP recognizing T cells are maintained or expanded in the tolerizing gut environment through recognition of cross-reactive microbiota HSPs. Several of the following factors could add to the efficiency of this immune imprinting of HSP reactivity at the Treg level. When microbes are being taken up by macrophages or DCs lining the gut, phagocytosis and exposure of the ingested bacteria to the hostile intracellular environment of the phagocyte will lead to a microbial HSP upregulating stress response (13). In addition, the continuous contact with a variable set of microbiota-associated bacterial species may emphasize the driving nature of just the conserved and therefore repeatedly encountered bacterial sequences. Similarly, stress in host tissues as seen in inflammation will also lead to the enhanced expression of self-HSPs and thereby enhance attractiveness as a target for T cell regulation.

**Table 1:** Mechanistic sequence of events leading to anti-inflammatory activity of heat shock protein (HSP)-specific regulatory T cell (Tregs).

(1) HSP expression in thymic epithelial cells (2) Loading of HSP peptides into MHC class II of positively selecting thymic epithelial cells (3) Repertoire of HSP-specific Tregs expanded and maintained by cross-recognition of conserved microbial (microbiota) HSP peptides in the gut (4) HSP overexpression due to inflammation (stress) in tissues (5) Selective targeting of HSP-specific Tregs to inflamed tissues

In the next sections, we will explain how the anti-inflammatory effects of HSPs can work in synergy with the cell therapy approach with tolDC.

## Different Approaches to Induce tolDCs and Their Function in Experimental Models

Experimental disease models have been used to explore the abilities of tolDCs to induce Tregs and their possible therapeutic application *in vivo* (14–16). Among these studies, tolDCs were also tested in an arthritis model. In this study, tolDCs were generated *in vitro* from murine bone marrow with dexamethasone and 1α,25-dihydroxyvitamin D3 (the active form of vitamin D3). Subsequently, they were pulsed with collagen type II (17). These tolDCs, showing a semi-mature phenotype, were able to reduce T cell proliferation and diminish arthritis severity. Unpulsed tolDCs were not able to reduce arthritis. This suggests that antigen is needed to suppress disease. Choosing the right antigen is important since most autoimmune diseases are caused by a deviant lymphocytic response against a self-antigen. Whereas rheumatoid arthritis (RA) affects one body component (the joint), other autoimmune diseases such as systemic lupus erythematosus (SLE) affect multiple organs. Can tolDC therapy also be applied in these type of autoimmune diseases? And could we induce tolDCs *in vivo* if the autoantigen is unknown?

Systemic lupus erythematosus is a multisystem autoimmune disease in which autoantibodies play an important role. Another feature of SLE is dysregulation of DCs as these cells continuously display a mature phenotype with high expression of costimulatory molecules and chemokine receptors (18, 19). Because dysregulated DCs are involved in the pathogenesis of SLE, DC therapy could contribute to the welfare of SLE patients. Monocyte-derived DCs (moDCs) from SLE patients were isolated as a first step in investigating the possibility of tolDC therapy. The moDCs were stimulated with iC3b-opsonized apoptotic cells or the combination of dexamethasone and vitamin D3. In both cases, the DCs gained tolerogenic properties (20, 21). This indicates that SLE DCs can be modified. Up until now, no *in vivo* studies have been performed with *in vitro* generated tolDCs in SLE but blocking NF-κB activity in DC-induced tolerogenic characteristics. SLE mice treated with these NF-κB blockers showed a reduction in circulating autoantibodies (22). These results suggest that inducing tolDCs *in vivo* could be a solution in SLE. The same group showed that this method is also successful in experimental immune encephalomyelitis (EAE) (23). Antigen-specific effects of NF-κB activity-blocked DCs in EAE were studied with MOG as the autoantigen. In this model also bone marrow-derived tolDCs loaded with MOG peptide (40–55) were found to reduce disease by the induction of Treg (15).

Next to using NF-κB blockers in SLE and EAE, they have also been used to generate tolDCs *in vitro* with regard to RA. More specifically, addition of the NF-κB inhibitor Bay11-7082 to bone marrow- or peripheral blood-derived DCs caused a lower expression of costimulatory molecules and weak stimulation of T cells (24, 25). These DCs, when pulsed with antigen and injected into mice, attenuated inflammatory arthritis via the induction of Tregs. To test if this could also be achieved *in vivo* without modulating DCs *in vitro*, solely liposomes containing antigen and a NF-κB inhibitor were infused into arthritic mice. Arthritic symptoms were reduced only when the antigen was codelivered with the NF-κB inhibitor. Merely the delivery of antigen or NF-κB inhibitor did not reduce arthritis (26). This shows that liposomes carrying both antigen and NF-κB inhibitor can target DCs *in vivo* to induce antigen-specific tolerance.

Other drug delivery systems have also been used to influence the status of a DC *in vivo*. DC stimulation with intranasal antigen encapsulated by polylactic-*co*-glycolic acid (PLGA) nanoparticles resulted in increased antigen uptake of the DCs and induction of CD4^+^FoxP3^+^ cells. Furthermore, PLGA nanoparticle treatment was tested in a delayed type hypersensitivity (DTH) model and an arthritis model. In the DTH model, the mice were treated with PLGA nanoparticles or control and subsequently sensitized with ovalbumin (OVA) in combination with incomplete Freund’s adjuvant and after 24 h challenged with OVA. PLGA nanoparticle treatment resulted in a reduced sensitivity reaction, whereas the controls did not (27). Next to this, nasal application with PLGA nanoparticles encapsulating mB29a (a mammalian HSP70 peptide) reduced arthritis severity for 30 days after disease development, suggesting that chronic inflammatory responses can also be modulated by tolDC (27). All in all, substantial evidence has been collected in preclinical models for an effective tolerance promoting effect of tolDC in autoimmunity.

## A Treg-Inducing HSP70 Peptide

Since the autoantigen in many autoimmune diseases is unknown, surrogate autoantigens could be used to restore tolerance. Among mammalian HSP, the HSP70 family of proteins contains some of the most stress-inducible HSPs, besides constitutive family members. In addition, some HSP70 family members are involved in chaperone-mediated autophagy (CMA). CMA contributes to maintenance of cellular homeostasis by facilitating recycling of degraded proteins and by eliminating abnormal or damaged proteins. HSP70, in combination with HSP90, is responsible for the targeting of proteins to the lysosome during CMA. MHC class II elution studies have shown that autophagy, as a consequence of cell stress, contributes to preferential loading of MHC II with HSP70 peptides (28). In the latter study, nutrient-deprived human HLA-DR4^+^ B cells were used for the analysis. Among the more abundant peptides present in the elution profile of the stressed B cells, there was a peptide that had been previously discovered by us as a dominant T cell epitope in Balb/c mice previously (29). This peptide was discovered, when immunizations with mycobacterial HSP70 were found to protect against disease in the proteoglycan-induced arthritis (PGIA) model. This particular peptide, called B29, triggered disease protective T cell responses and consisted of a highly conserved sequence (29). This mycobacterial HSP70-B29 peptide had mammalian homologs (counterparts), called mB29a, mB29b, and mB29c in both constitutive and stress-inducible HSP70 family members. Exactly the mB29b variant was present in the elution profile of these HLA-DR4^+^ B cells. Interestingly enough, the same mB29b variant was also reported to be present in the MHC-II clefts of human thymic antigen-presenting cells (12).

Our interest in the B29 peptides developed from the observations that nasal application of the peptide-suppressed PGIA in mice (29). Follow-up experiments made clear that B29 and its mammalian homologs were capable of inducing Tregs. Immunizations with B29 or an ovalbumin peptide (pOVA) as a control were performed, and CD25^+^ T cells were sorted by FACS from the responding CD4^+^ T cell population. Adoptive transfer of these CD25^+^ T cells led to reduction of PGIA in recipients, whereas CD25^−^ T cells did not. Also CD25^+^ and CD25^−^ populations obtained from pOVA immunized animals were not having any effect on arthritis. The cell numbers needed for reaching these effects were relatively low: 3 × 10^5^ cells sufficed for prevention of disease by adoptive transfer prior to disease induction, whereas only 1 × 10^6^ T cells were needed to suppress ongoing disease.

By the use of a congenic T cell marker (CD90.1), exclusively present on the transferred T cells, we were able to track the transferred T cells *in vivo* (13). Our transferred, disease suppressing, T cells were still found present 50 days after transfer in the spleen, draining lymph nodes, joints, bone marrow, and blood. In addition, they were found to have kept their Treg phenotype, as they expressed CD25, Foxp3, NRP-1 (neuropilin 1), and lymphocyte activation gene-3 (LAG-3). When we infused a depleting CD90.1 antibody during the phase of disease suppression, after transfer of the CD25^+^ T cells from B29 immunized donors, disease relapsed, reaching a severity identical with that in CD25^−^ T cell-transferred animals. Altogether, these experiments showed the potential of conserved HSP70 peptides to induce a Treg response, which is long-lived and actively engaged in suppression of disease. When we sorted the LAG-3 positive CD4^+^ T cells from our CD25^+^ population and transferred these cells before induction of PGIA, we prevented induction of disease with the very small number of 4,000 CD4^+^ T cells (29). As far as we know, this has never been seen in mouse models before, and it may indicate the superiority of antigen-selected Tregs as compared to non-antigen-selected Tregs. Furthermore, it also shows the strong potential of targeting Tregs to HSPs since the pOVA-induced Tregs never suppressed disease. Herewith, the example of HSP70 peptide B29 shows the potential of HSP peptides to effectively modulate immunity by the induction of Tregs.

## HSP-Mediated DC Modulation

Given the protective effects of mycobacterial HSP70 in arthritis models, the immune modulatory activities of this molecule were also studied in RA. Bonorino and her group have demonstrated that mycobacterial HSP70 is capable of inducing IL-10 production in cells obtained from the inflamed synovium of arthritis patients. In addition, this was found in peripheral blood mononuclear cells from RA patients and healthy controls. Besides this, TNF-α and INF-γ production in these cells decreased and IL-10 production was raised. Cell-separation studies showed that the cells that produced IL-10 were monocytes (30).

When mouse bone marrow-derived DC were exposed to mycobacterial HSP70, maturation markers MHC class II and CD86 remained suppressed, indicating a tolerogenic phenotype (31, 32). Also in the presence of lipopolysaccharide (LPS), HSP70 reduced the upregulation of these markers. As in human cells, mycobacterial HSP70 induced IL-10 and not TNF-α. Altogether, it was concluded that DC maturation was halted by mycobacterial HSP70. Furthermore, LPS-free mycobacterial HSP70 was seen to inhibit phytohemagglutinin-induced T cell proliferation and was not seen to induce CD86 expression on splenic DCs *in vivo*, whereas LPS did (31). Although various alternative receptors were claimed to act as cellular receptors for HSP70, the signaling leading to IL-10 production in these studies may have involved TLR2 triggering with MyD88 activation and ERK phosphorylation (33).

When HSP70-treated DCs were tested in the proteoglycan (PG)-induced arthritis model, suppression of disease induction was seen when the treated DCs were loaded in addition with PG (32). And interestingly, when OVA-specific (TCR transgenic) T cells were cotransferred together with OVA-pulsed HSP70-treated DCs, the OVA-specific T cells were producing increased levels of IL-10 when re-stimulated *in vitro* with OVA. This regulatory cytokine induction in DCs was seen for both mycobacterial and mammalian HSP70 (32).

Thus, it may be concluded that part of the tolerance-promoting effects of HSP is mediated through their capacity to induce a regulatory mode in DCs.

## Coinduction of Endogenous HSPs

Cell stress, caused by environmental factors or endogenous factors such as accumulation of unfolded proteins in the cytosol, is the primary trigger for upregulation of endogenous HSPs in cells. Such upregulation of HSPs lead, among others as the result of stress induced autophagy as discussed earlier, to further routing of HSP peptides to the MHC class II-binding grooves for recognition by T cells.

One possibility for enhancing HSP expression during stress is through the application of the so-called HSP coinducers. These are compounds that help to enhance production of HSP during stress, but are not capable of initiating a stress response on their own. When heat shock factor has become activated, this transcription factor induces the HSP synthesis, and coinducers just boost the level of production, through as yet unknown mechanisms.

A first and well-studied HSP coinducer, which is rather selective for the coinduction of HSP70 and not the others, is geranylgeranylacetone (GGA), an acyclic polyisoprenoid. Originally, GGA was developed as an effective anti-gastric ulcer drug. It has been tested now in various inflammatory diseases, including experimental autoimmune uveoretinitis (34). In this model, oral GGA inhibited disease and local HSP70 mRNA expression in the eyes was transiently upregulated. The autoantigen-specific T cell proliferation was also suppressed in GGA-treated mice (34).

A more recent example of an effective HSP coinducer is carvacrol, an essential oil present in Oregano species. Carvacrol was known to have antibacterial activity but upon testing on mammalian cells, carvacrol was found to have strong stress protein coinducing capacity (35). Carvacrol promoted HSP70 expression in human cell lines and mouse spleen cells during stress *in vitro* (caused by a raised temperature or exposure to arsenite). Upon intragastric administration, it resulted in raised HSP70 gene expression in Peyer’s patches of mice *in vivo*. As a consequence, the same intragastric administration of carvacrol specifically promoted T cell recognition of endogenous HSP70, as demonstrated by amplified T cell responses to HSP70. It also systemically increased the number of CD4^+^CD25^+^FoxP3^+^ T cells in the spleen, and almost completely suppressed PG-induced experimental arthritis.

Very similar to what was seen for HSP-treated DCs loaded with PG in the PGIA model, as mentioned in the previous section, heat stress (42.5°C for 1 h) and carvacrol treated DCs loaded with PG also protected against PGIA. In addition, these DCs induced expansions of Foxp3^+^ cells *in vivo* (17).

Herewith, it can be concluded that apart from the loading of DCs with HSP peptides, it would be possible in theory to use upregulated cell-endogenous HSPs for getting HSP peptides presented in tolDCs.

## The Attractive Possibility of HSP Peptide-Loaded tolDCs

Anti-inflammatory interventions using antigen-loaded tolDCs are already being developed for several chronic inflammatory diseases, such as diabetes type I and RA. First clinical trials indicated safety and have suggested clinical benefits (36, 37). In these cases, presumed relevant autoantigens were used to load the DCs.

In multiple sclerosis (MS), tolerogenic moDCs from relapsing-remitting (RR) MS patients, loaded with myelin peptides as specific antigen, were studied. The RR-MS tolDCs expressed a stable semi-mature phenotype and induced a stable antigen-specific hyporesponsiveness in myelin-reactive T cells from RR-MS patients *in vitro* (38).

A recent clinical trial in RA has attempted to induce tolerance for self-antigens present in the synovial fluids of inflamed joints (37). Nevertheless, the target group here was patients with active disease, which may have hindered the chances for real tolerance induction. In addition, not a well-defined antigen for immune monitoring was available in this study.

**Figure 1:**
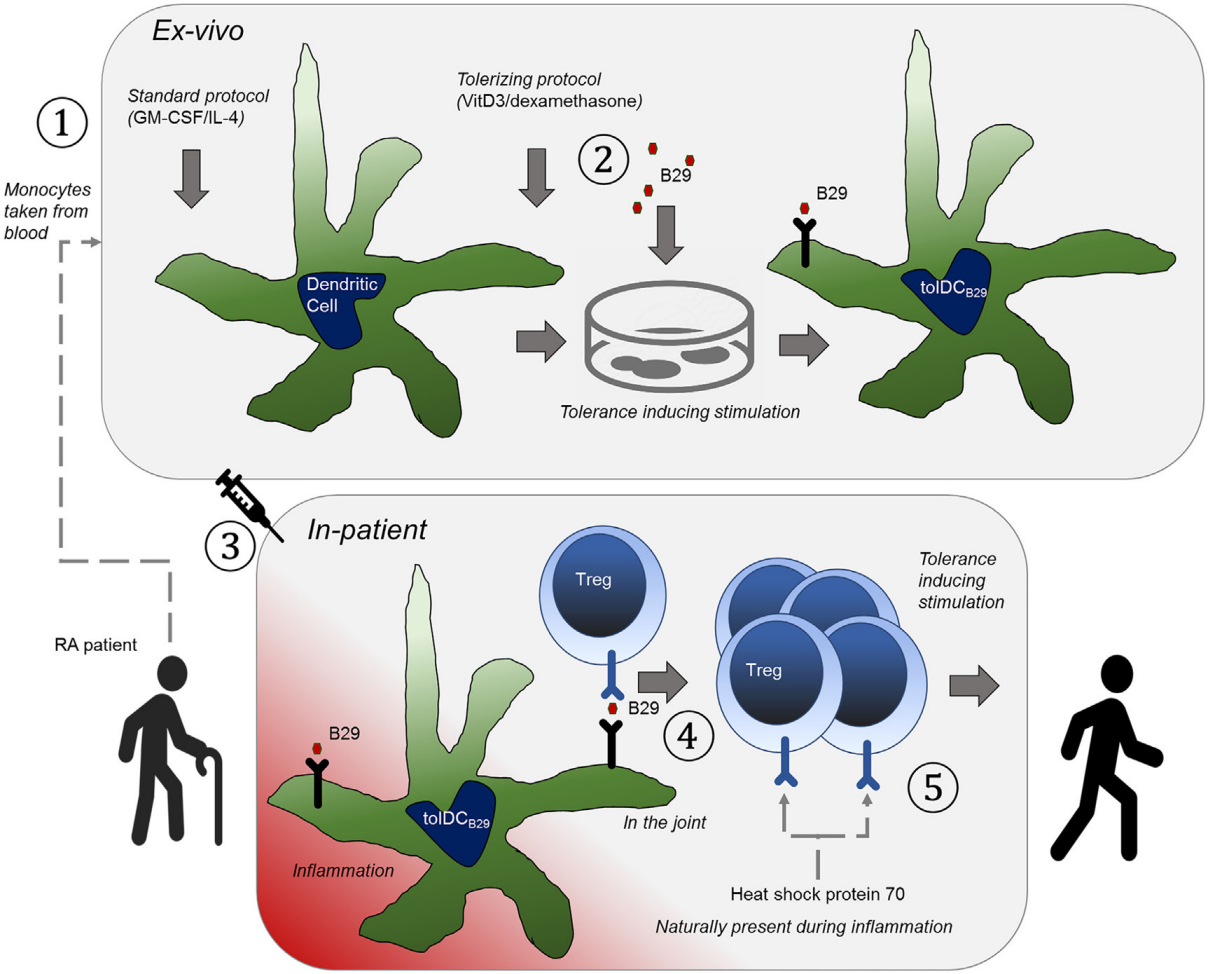
The basic principle that underpins the therapy is to educate tolerance-invoking regulatory T-cells. An orchestrated series of events needs to happen to achieve this and hence to allow it to be an effective approach to treatment of rheumatoid arthritis. (1) Tolerogenic heat shock protein (B29)-specific regulatory T cells (Tregs) are present in the patient, which can be verified with antigen-specific T cell assays. (2) The patient is treated with anti-TNF (or similar) to induce a state of disease remission. (3) Dendritic cells (DCs) are obtained from the patient by expanding peripheral blood obtained monocytes with GM-CSF/IL-4 as growth factors ①, (4) The DCs are *ex vivo* made into tolerogenic DCs with vitamin D3/dexamethasone and loaded with B29 ②, so that they can present this epitope to regulatory T cells. (5) The cells are re-introduced into the patient ③ (remission allows for better tolerance induction). (6) The epitope is presented to Tregs by the tolerogenic DCs ④, to activate the regulatory T cells. (7) The patient now has a Treg repertoire that naturally suppresses inflammation ⑤ (in the joint).

In the case of HSP-loaded tolDCs (see Figure 1), the use of a well-defined HSP antigen will help the exact monitoring of induced HSP-specific Tregs with defined specificity in clinical experiments. In addition, patients can be selected on the basis of their antigen-specific response profiles for inclusion in the trial. Given the fact that molecules such as HSP70 are upregulated in inflamed tissues, therapeutic tolerance can be achieved through the induction of HSP70-specific Tregs, which then *via* bystander suppression reduce inflammation irrespective of the disease or inciting autoantigens. Therefore, when shown to be effective, HSP peptide-loaded tolDCs could be of use for therapies directed toward inflammatory diseases in the broadest possible sense.

## Author Contributions

WE wrote most of the paper. MJ wrote part of the paper. IL, CW, PL, and FB were essential for discussing the content of the paper.

## Conflict of Interest Statement

WE and PL have shares in Trajectum Pharma. The co-authors declare that the research was conducted in the absence of any commercial or financial relationships that could be construed as a potential conflict of interest.
